# Characteristics of Walkable Built Environments and BMI *z*-Scores in Children: Evidence from a Large Electronic Health Record Database

**DOI:** 10.1289/ehp.1307704

**Published:** 2014-09-23

**Authors:** Dustin T. Duncan, Mona Sharifi, Steven J. Melly, Richard Marshall, Thomas D. Sequist, Sheryl L. Rifas-Shiman, Elsie M. Taveras

**Affiliations:** 1 Department of Social and Behavioral Sciences, Harvard School of Public Health, Boston, Massachusetts, USA; 2 Division of General Academic Pediatrics, Department of Pediatrics, Massachusetts General Hospital for Children, Boston, Massachusetts, USA; 3 Department of Environmental Health, Harvard School of Public Health, Boston, Massachusetts, USA; 4 Harvard Vanguard Medical Associates, Boston, Massachusetts, USA; 5 Division of General Medicine and Primary Care, Brigham and Women’s Hospital, Boston, Massachusetts, USA; 6 Department of Nutrition, Harvard School of Public Health, Boston, Massachusetts, USA

## Abstract

Background: Childhood obesity remains a prominent public health problem. Walkable built environments may prevent excess weight gain.

Objectives: We examined the association of walkable built environment characteristics with body mass index (BMI) *z*-score among a large sample of children and adolescents.

Methods: We used geocoded residential address data from electronic health records of 49,770 children and adolescents 4 to < 19 years of age seen at the 14 pediatric practices of Harvard Vanguard Medical Associates from August 2011 through August 2012. We used eight geographic information system (GIS) variables to characteri*z*e walkable built environments. Outcomes were BMI *z*-score at the most recent visit and BMI *z*-score change from the earliest available (2008–2011) to the most recent (2011–2012) visit. Multivariable models were adjusted for child age, sex, race/ethnicity, and neighborhood median household income.

Results: In multivariable cross-sectional models, living in closer proximity to recreational open space was associated with lower BMI *z*-score. For example, children who lived in closest proximity (quartile 1) to the nearest recreational open space had a lower BMI *z*-score (β = –0.06; 95% CI: –0.08, –0.03) compared with those living farthest away (quartile 4; reference). Living in neighborhoods with fewer recreational open spaces and less residential density, traffic density, sidewalk completeness, and intersection density were associated with higher cross-sectional BMI *z*-score and with an increase in BMI *z*-score over time.

Conclusions: Overall, built environment characteristics that may increase walkability were associated with lower BMI *z*-scores in a large sample of children. Modifying existing built environments to make them more walkable may reduce childhood obesity.

Citation: Duncan DT, Sharifi M, Melly SJ, Marshall R, Sequist TD, Rifas-Shiman SL, Taveras EM. 2014. Characteristics of walkable built environments and BMI *z*-scores in children: evidence from a large electronic health record database. Environ Health Perspect 122:1359–1365; http://dx.doi.org/10.1289/ehp.1307704

## Introduction

Childhood obesity is a prominent public health problem affecting millions around the globe, including U.S. children and adolescents (Ogden et al. 2012). A variety of built environment characteristics can increase child physical activity (e.g., utilitarian physical activity, leisure physical activity, and exercise), which, in turn, can decrease childhood obesity (Bennett et al. 2008; Cradock and Duncan 2014). Indeed, built environment characteristics can influence different physical activity outcomes: Parks may be associated with leisure-time physical activity, and land use mix may be associated with transport physical activity (Ding et al. 2011; Ferdinand et al. 2012). Therefore, walkable built environments—herein defined as everything made or maintained by people with characteristics that can promote increases in physical activity—may prevent excess weight gain and reduce obesity prevalence among children (Cradock and Duncan 2014).

Built environments are recognized in the White House Task Force Report on Childhood Obesity as an important contributor to childhood obesity (Executive Office of the President of the United States 2010). However, the large and complex literature on the effects of the built environment on childhood obesity outcomes has been mixed in terms of the significance, direction, and magnitude of effect (Dunton et al. 2009; Feng et al. 2010). For example, systematic reviews have documented that some studies estimated statistically significant associations between indicators of walkable built environments and childhood obesity, but others found no significant associations (Dunton et al. 2009; Feng et al. 2010). The literature on the built environment and childhood obesity is subject to several addressable limitations, particularly small sample sizes and temporal ambiguity (Dunton et al. 2009; Feng et al. 2010). Relatively small sample sizes could lead to inadequate power to estimate statistically significant associations, when they exist. Additionally, longitudinal study designs are important for causal inference, by providing a clear temporal ordering and the ability to take into account (potential) time lags between exposure to built environment characteristics and body weight change. Study designs that incorporate and account for lag times are important, especially for an outcome such as body mass index (BMI)—compared with behavioral outcomes such as physical activity, which likely respond to changes in exposures more rapidly (Diez Roux 2007). The few existing longitudinal studies examining relationships between built environments and BMI among children and adolescents have had inconsistent results, with some reporting statistically significant longitudinal associations (Bell et al. 2008; Jerrett et al. 2010; Wolch et al. 2011) but not others (Crawford et al. 2010; Ewing et al. 2006; Gose et al. 2013), perhaps owing to relatively small sample sizes in some of these studies. Another notable limitation of much of the previous built environment–childhood obesity research is the use of self-reported BMI information (Dunton et al. 2009), which can be subject to misclassification. In particular, use of self-reported BMI can result in differential misclassification—which can lead to overestimation or underestimation of the true association (Rothman et al. 2008).

The purpose of the present study was to estimate associations of walkable built environment characteristics with BMI *z*-scores based on data from a large sample of children and adolescents included in an electronic health record database that has objective measures of BMI, thus addressing the aforementioned limitations of existing research. In addition to cross-sectional analyses of BMI *z*-score, we analyzed change in BMI *z*-score as an outcome, regressed on walkable built environment characteristics (our secondary study aim). We hypothesized that indicators of neighborhood walkability would be associated with lower BMI *z*-scores cross-sectionally, and that characteristics expected to reduce walkability would be associated with increases in BMI *z*-scores over time among children and adolescents, based on past theoretical and empirical research. This suggests that walkable built environments can promote increased energy expenditure (Bennett al. 2008; Cradock and Duncan 2014; Ding et al. 2011; Ferdinand et al. 2012).

## Materials and Methods

**Figure 1:**
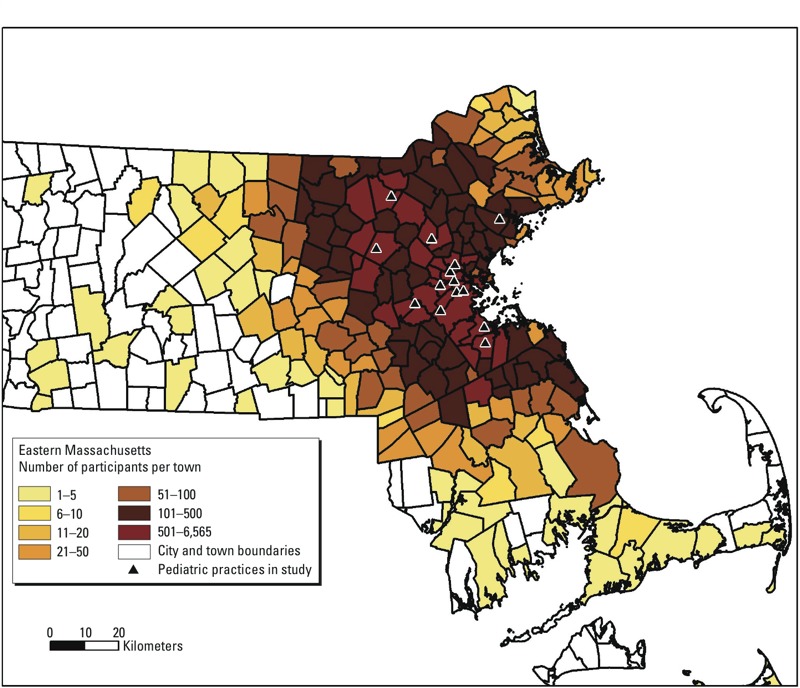
Number of participants by town in Massachusetts, *n* = 49,770.

*Study design and participants*. We used geocoded residential address data from the electronic health records of children and adolescents from 14 pediatric practices of Harvard Vanguard Medical Associates, a large multi-site, multi-specialty physician group practice in Massachusetts. Figure 1 shows the number of participants by town. Inclusion criteria for the study varied by our two study aims; for aim 1 (cross-sectional analyses), the criteria for inclusion were *a*) having a residential address in Massachusetts; *b*) child was between the ages of 4 to < 19 years; *c*) at least one BMI *z*-score was available from a well-child visit between August 2011 and August 2012; *d*) there were no outlier BMI *z*-score values (< 6 and > –6) based on the Centers for Disease Control and Prevention (CDC) 2000 reference data (Kuczmarski et al. 2002); and *e*) the child/adolescent had no known pregnancy and had no chronic medical condition (i.e., leukemia and inflammatory bowel disease) that might have affected growth and nutrition. The final analytic sample size for the cross-sectional aim was 49,770. For aim 2 (longitudinal analyses), we additionally required *f*) availability of at least two BMI measures between January 2008 and August 2012 and *g*) ≥ 1 year between the first and last BMI measurement. The sample size for our longitudinal analyses was reduced to 46,813. The Institutional Review Board of Harvard Pilgrim Health Care approved the study protocol. In particular, we received a waiver of informed consent for this study. Because of the number of medical records needed for review, approximately 80,000, requesting informed consent from each individual would be extremely burdensome and could potentially skew the sampling due to bias of response. The research could not be conducted without the participants’ address(es) because these were essential for the process of geocoding.

*Exposures: walkable built environment characteristics*. Using the most recent residential addresses of the children (at time of BMI for cross-sectional analysis), we used eight geographic information system (GIS) variables to characterize walkable built environments. Increased access to recreational open space may be associated with leisure-time physical activity among children, through walking to and playing at parks (Ding et al. 2011). Children might also see others being physically active if the open space contributes to neighborhood esthetics that encourage physical activity. Therefore, we measured network distance to nearest public and private recreational open space (e.g., parks, playing fields, school fields) in kilometers (up to a maximum of 15 km, which is almost 9.5 miles) using data from the Office of Geographic Information (MassGIS), Commonwealth of Massachusetts, Information Technology Division (http://www.mass.gov/anf/research-and-tech/it-serv-and-support/application-serv/office-of-geographic-information-massgis/). We also used MassGIS data to derive a count of public and private recreational open space variable using an 800-m line-based network buffer (data from MassGIS). More housing units (which are regulated by such mechanisms as zoning) could be associated with more child physical activity, because children might see others being physically active and be able to walk to homes of other children their age (Ding et al. 2011). Therefore, we measured residential density (number of housing units per square kilometer) for the census tract [data from 2006–2010 American Community Survey (U.S. Census Bureau 2010)]. High traffic density in the immediate vicinity of homes could inhibit physical activity because busy roads might be a physical barrier and parents and youth might not feel safe walking in high traffic density areas (Ding et al. 2011). Therefore, we measured traffic density in 800-m line-based network buffers (average daily traffic × length of roads in meters in 800-m line-based network buffers divided by buffer area in square kilometers) (data from Massachusetts Department of Transportation, Boston, MA). Higher neighborhood speed limit could reduce the walkability of a neighborhood (Ding et al. 2011). Arteries and highways (which have higher speed limits and are noisy) are potential local physical barriers or perceived safety hazards. Therefore, we measured average speed limit (average speed limit of StreetMap 10 road segments that intersect 800-m line-based network buffers weighted by the length of road within the buffer) (data from ArcGIS, version 10, StreetMap; ESRI, Redlands, CA). More sidewalks could result in more socialization, play, and walking among children (Ding et al. 2011). Therefore, we measured sidewalk completeness within 800-m network buffers calculated for roads without medians (0 = no sidewalks, 1 = sidewalk on one side, 2 = sidewalks on both sides on all road segments in buffer) (2009 data from the Massachusetts Department of Transportation). More intersections could offer more direct route choices (Krizek 2003) and result in more walking in the local neighborhood including to local stores and parks (Ding et al. 2011). Therefore, we measured intersection density (intersections per square kilometer in 800-m line-based network buffer; intersections defined as points where three or more road segments come together, using the pedestrian street network) (data from ESRI ArcGIS, version 10, StreetMap). Finally, land-use mix may be associated with transport physical activity: greater variety of land use or services available near one’s home could result in more children walking (Ding et al. 2011). Therefore, we measured land use mix, using 2009 data from ESRI Network Analyst Extension, version 10.1. Land use mix was calculated using business point locations for five types of businesses (food, retail, services, cultural/educational, physical activity) whereby a land use mix of 0 corresponds to no variety of businesses, and 1 indicates businesses that are an equal mixture of all five categories. We specifically selected built environment indicators that have been theoretically and/or empirically associated with child physical activity or BMI in previous research and that are potentially modifiable.

We selected the 800-m egocentric street network buffer not only to minimize spatial misclassification (Duncan et al. 2014), but because this distance is relevant for children and adolescents (Colabianchi et al. 2007; Timperio et al. 2004), street networks are relevant to human movement patterns (Boruff et al. 2012; Oliver et al. 2007), and this neighborhood definition has been used in previous built environment–youth BMI research (Duncan et al. 2012; Gose et al. 2013; Kligerman et al. 2007; Potwarka et al. 2008; Schwartz et al. 2011). We created the 800-m line-based network buffer, using the ArcGIS 10.1 Network Analyst Extension Areas within 50 m of street center lines were included in the buffers. For analyses, we categorized each walkable built environment variable into quartiles.

*Outcomes: BMI* z*-score and change in BMI* z*-score.* Given the variation in ages that the population includes and the various levels of age- and sex-specific adiposity, we examined BMI *z*-score as our outcomes. The outcome for our cross-sectional analysis was BMI *z*-score at the child’s most recent well-child visit from August 2011 through August 2012; for our longitudinal analysis, the outcome was change in BMI *z*-score from the child’s first available BMI measure between January 2008 and August 2011 to the most recent BMI measure between August 2011 and August 2012. At each well-child visit, medical assistants measured height and weight based on the written standardized protocol of the health centers, which are consistent with the standard of care in pediatric primary care. We calculated BMI as kilogram per meter squared and used the CDC growth curves to define the participant’s age- and sex-specific BMI *z*-score (Kuczmarski et al. 2002).

*Other variables.* We collected individual-level data on date of birth, sex, date of the well-child visit, and parent report of child’s race/ethnicity from the electronic health records. We also collected data on neighborhood median household income at the census tract level from the 2006–2010 American Community Survey (U.S. Census Bureau 2010).

*Statistical analysis.* First, we calculated descriptive statistics of the study sample and used Spearman correlations to examine the bivariate relationships between the measures of walkable built environments and BMI *z*-score. We then conducted the cross-sectional analyses of the associations of the GIS walkable built environment variables with BMI *z*-score. For these cross-sectional associations, we examined unadjusted and multivariate adjusted models. Model 1 was unadjusted. In model 2, we adjusted for age, sex, and race/ethnicity (black, Hispanic, Asian, white, and other in addition to a missing category). In model 3, we adjusted for variables in model 2 and added neighborhood median household income (continuous). We also analyzed the associations of the GIS walkable built environment variables with change in BMI *z*-score. For the change analyses, we adjusted for the variables listed above as well as for change in age from baseline to follow-up (i.e., months between the first available BMI measure to the most recent). In a sensitivity analysis, we subsequently computed model 3 by quartile (Q) of neighborhood household median income (mean Q1 = $48,435; mean Q2 = $72,626; mean Q3 = $93,327; and mean Q4 = $132,060). In exploratory analyses, we used stratified models and also ran interaction *p*-values to examine potential effect modification by age in categories (4 to < 10, 10 to < 14, 14 to < 19 years), sex (male, female) and race/ethnicity (black, Hispanic, Asian, white, and other in addition to a missing category). Some of the interaction *p*-values were *p* < 0.05 (likely because of the very large sample si*z*e). Overall, there was no or minimal evidence of effect modification (i.e., results look similar across strata), so for parsimoniousness, we present aggregate models. In all models, we calculated *p*-values for trends across quartiles. For trend *p*-values across quartiles, we included quartiles as an ordinal variable, coded as 1-2-3-4. We analyzed the impact of one built environment variable on BMI at a time, partly due to collinearity between built environments shown in previous research (Leal et al. 2012). Statistical analyses were conducted in SAS version 9.3 (SAS Institute Inc., Cary, NC). Statistical significance was determined by *p* < 0.05.

## Results

Table 1 shows the characteristics of the 49,770 children and adolescents included in the study. The average neighborhood median yearly household income was $86,577 ± $33,196. The mean (± SD) age of children included in the cross-sectional analysis was 11.3 ± 4.4 years, and the mean BMI *z*-score at the most recent well-child visit was 0.42 ± 1.04. Approximately 28% of the children had a BMI ≥ 85th percentile. The mean age change from the first to last visit was 3.2 ± 0.8 years, and the mean BMI *z*-score change was 0.07 ± 0.7 units.

**Table 1:** Characteristics of children and adolescents 4 to < 19 years of age seen for a well-child visit (*n* = 49,770).

Characteristic	Mean ± SD or *n* (%)
Neighborhood characteristics
Census tract median household income/year	$86,577 ± $33,196
Child characteristics
Sex
Male	25,153 (50.5)
Female	24,617 (49.5)
Race/ethnicity
Black	5,943 (11.9)
Hispanic	2,549 (5.1)
Asian	4,166 (8.4)
White	29,206 (58.7)
Other	2,946 (5.9)
Missing	5,943 (11.9)
First visit (earliest)^*a*^
Age (years)	8.1 ± 4.3
BMI *z*-score	0.36 ± 1.09
Last visit (most recent)
Age (years)	11.3 ± 4.4
BMI *z*-score	0.42 ± 1.04
Change first to last visit^*a*^
Age (years)	3.2 ± 0.8
BMI *z*-score	0.07 ± 0.7
^***a***^*n* = 46,813.	

Table 2 shows the distribution of walkable built environments characteristics. Table 3 shows correlations for the most recent BMI *z*-score and the walkable built environments characteristics. *p*-Values for all correlations, including correlations between the measures of walkable built environment characteristics and the correlations between built environments and BMI *z*-score, were significant at the < 0.0001 level, except for BMI *z*-score and count of recreational open space (*p* = 0.003) and BMI *z*-score and distance to nearest recreational open space (*p* = 0.09).

**Table 2:** Distributions of child/adolescent BMI *z*-score and characteristics of neighborhood walkability, *n* = 49,770.

Variable	Mean ± SD	Median (range)
BMI *z*-score^*a*^	0.42 ± 1.04	0.42 (–5.55, 5.81)
Recreational open space [distance (km)]	0.55 ± 0.54	0.40 (0, 14.65)
Recreational open space (count)	2.76 ± 2.98	2.00 (0, 22.00)
Residential density (per km^2^)	1,063 ± 1,531	456 (0–23,646)
Traffic density [(average daily traffic × length of road)/km^2^]	51,782,205 ± 47,509,629	41,639,588 (833,690–599,693,713)
Average speed limit (mph)	27 ± 2	27.0 (4.7–39.2)
Sidewalk completeness	0.87 ± 0.66	0.86 (0–2.00)
Intersection density (per km^2^)	78 ± 38	75.3 (0–319.5)
Land use mix	0.47 ± 0.35	0.65 (0–0.99)
mph, miles per hour. ^***a***^Last (most recent) BMI *z*-score.		

**Table 3:** Spearman correlation coefficients of child/adolescent BMI *z*-score and characteristics of neighborhood walkability.

Variable	BMI *z*‑score^*a*^	Recreational open space (distance)	Recreational open space (count)	Residential density	Traffic density	Average speed limit	Sidewalk completeness	Intersection density	Land use mix
BMI *z*-score	1.00								
Recreational open space [distance (km)]	0.01	1.00							
Recreational open space (count)	0.01	–0.60	1.00						
Residential density (per km^2^)	0.08	–0.31	0.66	1.00					
Traffic density [(average daily traffic × length of road)/km^2^]	0.04	–0.22	0.46	0.54	1.00				
Average speed limit (mph)	0.02	–0.09	0.22	0.21	0.57	1.00			
Sidewalk completeness	0.07	–0.31	0.64	0.82	0.50	0.19	1.00		
Intersection density (per km^2^)	0.06	–0.34	0.71	0.85	0.60	0.23	0.79	1.00	
Land use mix	0.04	–0.30	0.58	0.59	0.59	0.28	0.59	0.66	1.00
mph, miles per hour. All correlation coefficient *p*-values < 0.0001, except BMI *z*-scores and recreational open space (distance) *p* = 0.09, and BMI *z*-score and recreational open space (count) *p* = 0.003. ^***a***^Last (most recent) BMI *z*-score.									

In Table 4, we show associations of walkable built environment characteristics with BMI *z*-score. In unadjusted models (model 1) and models adjusted for child age, sex, and race/ethnicity, we found that several walkable built environment characteristics were associated with child BMI *z*-score. However, in several instances, the direction of the association changed in model 3 when we adjusted for neighborhood median income (e.g., the association of residential density with child BMI *z*-score). In these instances, models not adjusted for neighborhood median household income in associations of walkable built environment variables with BMI *z*-score are underadjusted, and neighborhood median household income is a qualitative confounder. In model 3, we found that those living in closest proximity (quartile 1) to nearest recreational open space had a lower BMI *z*-score (β = –0.06; 95% CI: –0.08, –0.03) compared with those living furthest away (quartile 4, reference). Children living in neighborhoods with fewer recreational open spaces, and less residential density, less traffic density, less sidewalk completeness, less intersection density, and less land use mix had a higher BMI *z*-score. For example, those living in quartiles 1 (i.e., fewest recreational open spaces) (β = 0.10; 95% CI: 0.07, 0.13), 2 (β = 0.13; 95% CI: 0.10, 0.15), and 3 (β = 0.08; 95% CI: 0.05, 0.10) of the count of recreational open space had a higher BMI *z*-score compared with those living in areas with the most recreational open spaces (quartile 4, reference). No associations were found between speed limit quartiles and BMI *z*-score in these multivariate models.

**Table 4:** Associations of neighborhood walkability in quartiles with most recent, cross-sectional BMI *z*-score [β (95% CI)], *n* = 49,770.

Exposure	Model 1	Model 2	Model 3
Nearest recreational open space (km)
0.0–0.2	–0.01 (–0.03, 0.02)	–0.02 (–0.05, 0.00)	–0.06 (–0.08, –0.03)*
0.2–0.4	0.02 (0.00, 0.05)	–0.01 (–0.03, 0.02)	–0.05 (–0.08, –0.03)*
0.4–0.7	0.06 (0.03, 0.08)*	0.03 (0.00, 0.05)	–0.01 (–0.03, 0.02)
0.7–14.6	0.0 (referent)	0.0 (referent)	0.0 (referent)
Trend *p*-value	0.12	0.02	< 0.0001
Recreational open space (count)
0	–0.05 (–0.07, –0.02)*	0.01 (–0.02, 0.04)	0.10 (0.07, 0.13)*
1	0.02 (–0.01, 0.05)	0.06 (0.03, 0.09)*	0.13 (0.10, 0.15)*
2–4	0.02 (–0.01, 0.05)	0.04 (0.01, 0.06)*	0.08 (0.05, 0.10)*
5–22	0.0 (referent)	0.0 (referent)	0.0 (referent)
Trend *p*-value	0.001	0.37	< 0.0001
Residential density (per km^2^)
0–174	–0.21 (–0.24, –0.19)*	–0.10 (–0.12, –0.07)*	0.11 (0.08, 0.14)*
174–456	–0.14 (–0.17, –0.12)*	–0.02 (–0.05, 0.01)	0.11 (0.08, 0.14)*
459–1414	–0.06 (–0.09, –0.04)*	0.01 (–0.02, 0.03)	0.09 (0.06, 0.12)*
1,416–23,646	0.0 (referent)	0.0 (referent)	0.0 (referent)
Trend *p*-value	< 0.0001	< 0.0001	< 0.0001
Traffic density [(average daily traffic × length of road)/km^2^]
833,690–23,138,768	–0.11 (–0.13, –0.08)*	–0.05 (–0.07, –0.02)*	0.03 (0.01, 0.06)*
23,138,903–41,638,769	–0.01 (–0.03, 0.02)	0.02 (0.00, 0.05)	0.05 (0.03, 0.08)*
41,639,588–64,141,476	0.02 (0.00, 0.05)	0.02 (0.00, 0.05)	0.03 (0.00, 0.06)
64,141,933–599,693,713	0.0 (referent)	0.0 (referent)	0.0 (referent)
Trend *p*-value	< 0.0001	0.0001	0.004
Average speed limit (mph)
4.7–26.0	–0.05 (–0.07, –0.02)*	0.00 (–0.03, 0.02)	0.03 (0.00, 0.05)
26.0–27.0	0.01 (–0.01, 0.04)	0.02 (–0.01, 0.04)	0.01 (–0.02, 0.03)
27.0–28.1	0.03 (0.01, 0.06)*	0.03 (0.00, 0.05)	0.01 (–0.02, 0.03)
28.1–39.2	0.0 (referent)	0.0 (referent)	0.0 (referent)
Trend *p*-value	0.0001	0.64	0.07
Sidewalk completeness
0.0–0.2	–0.19 (–0.22,–0.17)*	–0.09 (–0.12,–0.06)*	0.03 (0.00, 0.06)
0.2–0.9	–0.07 (–0.10,–0.05)*	0.01 (–0.02, 0.03)	0.08 (0.05, 0.10)*
0.9–1.5	–0.04 (–0.07,–0.01)*	0.01 (–0.01, 0.04)	0.04 (0.02, 0.07)*
1.5–2.0	0.0 (referent)	0.0 (referent)	0.0 (referent)
Trend *p*-value	< 0.0001	< 0.0001	0.01
Intersection density (per km^2^)
0.0–47.0	–0.19 (–0.21, –0.16)*	–0.09 (–0.12,–0.06)*	0.06 (0.03, 0.09)*
47.0–75.3	–0.07 (–0.10,–0.05)*	0.00 (–0.03, 0.03)	0.09 (0.07, 0.12)*
75.3–104.1	–0.06 (–0.09,–0.04)*	–0.02 (–0.04, 0.01)	0.04 (0.01, 0.07)*
104.1–319.5	0.0 (referent)	0.0 (referent)	0.0 (referent)
Trend *p*-value	< 0.0001	< 0.0001	< 0.0001
Land use mix
0.0–0.0	–0.09 (–0.12,–0.07)*	–0.05 (–0.07,–0.02)*	0.04 (0.02, 0.07)*
0.1–0.6	0.00 (–0.03, 0.03)	0.02 (–0.01, 0.05)	0.05 (0.02, 0.08)*
0.6–0.8	0.10 (0.07, 0.13)*	0.07 (0.05, 0.10)*	0.05 (0.03, 0.08)*
0.8–1.0	0.0 (referent)	0.0 (referent)	0.0 (referent)
Trend *p*-value	< 0.0001	< 0.0001	0.003
mph, miles per hour. For analyses, we categorized each walkable built environment variable into quartiles, so each built environment variable is divided into four equal groups. For example, those in quartile 1 of the nearest recreational open space have closer open spaces compared to quartile 4. Model 1: unadjusted; model 2: adjusted for age, sex, and race/ethnicity (with a missing category); model 3: model 2 + neighborhood median household income (continuous). *p < 0.05.			

Table 5 shows associations of walkable built environment characteristics with change in BMI *z*-score. In model 3 of the longitudinal analyses, we found that living in areas with the least amount of recreational open spaces, residential density, traffic density, sidewalk completeness, and intersection density was associated with an increase in BMI *z*-score over time (Table 5). We found no relationship between distance to recreational open spaces, speed limits, and land use mix with change in BMI *z*-score in these multivariate models.

**Table 5:** Associations of neighborhood walkability in quartiles with change in BMI *z*-score [β (95% CI)], *n* = 46,813.

Exposure	Model 1	Model 2	Model 3
Nearest recreational open space (km)
0.0–0.2	–0.01 (–0.02, 0.01)	–0.01 (–0.03, 0.00)	–0.02 (–0.03, 0.00)
0.2–0.4	0.01 (0.00, 0.03)	0.00 (–0.02, 0.01)	0.00 (–0.02, 0.01)
0.4–0.7	0.02 (0.00, 0.04)	0.01 (–0.01, 0.03)	0.01 (–0.01, 0.03)
0.7–14.6	0.0 (referent)	0.0 (referent)	0.0 (referent)
Trend *p*-value	0.41	0.04	0.03
Recreational open space (count)
0	–0.01 (–0.03, 0.01)	0.03 (0.01, 0.05)*	0.03 (0.01, 0.05)*
1	0.00 (–0.02, 0.02)	0.02 (0.00, 0.04)	0.03 (0.01, 0.05)*
2–4	0.00 (–0.01, 0.02)	0.02 (0.00, 0.04)	0.02 (0.01, 0.04)*
5–22	0.0 (referent)	0.0 (referent)	0.0 (referent)
Trend *p*-value	0.24	0.0056	0.0012
Residential density (per km^2^)
0–174	–0.04 (–0.06, –0.02)*	0.02 (0.00, 0.04)	0.04 (0.02, 0.06)*
174–456	–0.01 (–0.03, 0.00)	0.02 (0.00, 0.04)	0.03 (0.01, 0.05)*
459–1,414	–0.01 (–0.03, 0.01)	0.01 (–0.01, 0.03)	0.02 (0.00, 0.04)
1,416–23,646	0.0 (referent)	0.0 (referent)	0.0 (referent)
Trend *p*-value	< 0.0001	0.02	0.0002
Traffic density [(average daily traffic × length of road)/km^2^]
833,690–23,138,768	–0.02 (–0.04, –0.01)*	0.02 (0.00, 0.04)	0.02 (0.01, 0.04)*
23,138,903–41,638,769	–0.01 (–0.02, 0.01)	0.02 (0.00, 0.03)	0.02 (0.00, 0.04)
41,639,588–64,141,476	0.01 (–0.01, 0.03)	0.02 (0.00, 0.03)	0.02 (0.00, 0.03)
64,141,933–599,693,713	0.0 (referent)	0.0 (referent)	0.0 (referent)
Trend *p*-value	0.0028	0.04	0.01
Average speed limit (mph)
4.7–26.0	0.00 (–0.02, 0.01)	0.02 (0.00, 0.03)	0.02 (0.00, 0.04)
26.0–27.0	–0.01 (–0.03, 0.01)	0.00 (–0.02, 0.01)	0.00 (–0.02, 0.01)
27.0–28.1	0.00 (–0.02, 0.02)	0.00 (–0.02, 0.02)	0.00 (–0.02, 0.02)
28.1–39.2	0.0 (referent)	0.0 (referent)	0.0 (referent)
Trend *p*-value	0.4053	0.0954	0.0805
Sidewalk completeness
0.0–0.2	–0.01 (–0.03, 0.00)	0.03 (0.01, 0.05)*	0.04 (0.02, 0.06)*
0.2–0.9	0.00 (–0.02, 0.02)	0.02 (0.01, 0.04)*	0.03 (0.01, 0.05)*
0.9–1.5	0.01 (–0.01, 0.03)	0.02 (0.00, 0.04)	0.02 (0.01, 0.04)*
1.5–2.0	0.0 (referent)	0.0 (referent)	0.0 (referent)
Trend *p*-value	0.0818	0.0011	< 0.0001
Intersection density (per km^2^)
0.0–47.0	–0.03 (–0.05,–0.02)*	0.02 (0.00, 0.04)	0.03 (0.01, 0.05)*
47.0–75.3	–0.02 (–0.03, 0.00)	0.01 (0.00, 0.03)	0.02 (0.00, 0.04)
75.3–104.1	–0.01 (–0.03, 0.01)	0.01 (–0.01, 0.03)	0.01 (–0.01, 0.03)
104.1–319.5	0.0 (referent)	0.0 (referent)	0.0 (referent)
Trend *p*-value	0.0002	0.0444	0.0033
Land use mix
0.0–0.0	–0.03 (–0.04,–0.01)*	0.01 (–0.01, 0.02)	0.01 (0.00, 0.03)
0.1–0.6	–0.01 (–0.03, 0.01)	0.01 (–0.01, 0.03)	0.01 (–0.01, 0.03)
0.6–0.8	0.01 (–0.01, 0.02)	0.00 (–0.02, 0.02)	0.00 (–0.02, 0.01)
0.8–1.0	0.0 (referent)	0.0 (referent)	0.0 (referent)
Trend *p*-value	0.0005	0.26	0.09
mph, miles per hour. For analyses, we categorized each walkable built environment variable into quartiles, so each built environment variable is divided into four equal groups. For example, those in quartile 1 of the nearest recreational open space have closer open spaces compared to quartile 4. Model 1: unadjusted; model 2: adjusted for age, change in age baseline-follow-up, sex, and race/ethnicity (with a missing category); model 3: model 2 + neighborhood median household income (continuous). *p < 0.05.			

Some of the associations, in both the cross-sectional and longitudinal analyses, were not linear (*p* > 0.05 for trend *p*-values across quartiles, where we included quartiles as an ordinal variable, coded as 1-2-3-4). All models presented controlled for neighborhood median income as a continuous variable. When we controlled for neighborhood median income as a categorical variable, we observed similar results across models (data not shown).

We also performed sensitivity analyses where we stratified the results by quartiles of neighborhood median household income (mean Q1 = $48,435, mean Q2 = $72,626, mean Q3 = $93,327, and mean Q4 = $132,060), and the findings were generally consistent across all quartiles of median household income (see Supplemental Material, Tables S1 and S2). However, as shown in Table S1, in the cross-sectional results stratified by neighborhood median household income, significant associations seem to be isolated primarily in lower-income neighborhoods (i.e., Q1 and Q2).

## Discussion

Using electronic health record data comprising a large sample of children and adolescents in Massachusetts, we examined the relationship of walkable built environment characteristics with cross-sectional BMI *z*-score and change in BMI *z*-score over time. We found that living closer to recreational open space was associated with lower BMI *z*-score. Furthermore, living in areas with fewer recreational open spaces, less residential density, less intersection density, less land use mix, less traffic density, and less sidewalk completeness was associated with higher BMI *z*-score. We found no associations between living in areas with higher speed limit with BMI *z*-score. In longitudinal analyses, living in areas with less recreational open space, less residential density, less traffic density, less sidewalk completeness, and less intersection density was associated with an increase in BMI *z*-score over time. We found no relationship between distance to recreational open spaces, speed limits, and land use mix with change in BMI *z*-score.

Our study extends previous built environment research on BMI among children and adolescents by including a large sample from electronic health records and by analyzing change in BMI *z*-score as a longitudinal outcome. Other studies have examined built environment correlates of BMI among children and adolescents, with mixed findings (Dunton et al. 2009; Feng et al. 2010). A few studies have geocoded large samples of children and adolescents from electronic health records to examine built environment correlates of BMI (Liu et al. 2007; Oreskovic et al. 2009a, 2009b; Schwartz et al. 2011). For example, using electronic health record data with a sample of 7,334 children, Liu et al. (2007) found increased neighborhood vegetation was associated with decreased risk for childhood overweight. Using electronic health record data with a sample of 47,769 children, Schwartz et al. (2011) found that land use mix was associated with lower BMI, road density was associated with higher BMI, population density was associated with lower BMI, and county sprawl index was associated with higher BMI (Schwartz et al. 2011). These associations of neighborhood environmental measures differed by age (Schwartz et al. 2011). Our study did not find much evidence of age, sex, or race/ethnicity differences—based on comparable estimates across strata (data not shown) in estimated effects of the built environment on BMI, unlike some other studies (Alexander et al. 2013; Duncan et al. 2012).

Although few studies have had any longitudinal component in examining relationships between built environments and BMI among children and adolescents, some have been conducted. One study found no longitudinal association between traffic exposure and child BMI *z*-score (Crawford et al. 2010), whereas another found a positive longitudinal relationship between car traffic and attained BMI at 18 years of age (Jerrett et al. 2010). In cross-sectional analyses, Ewing et al. (2006) found an inverse association with county sprawl and adolescent BMI, but no association longitudinally. Although one study found no longitudinal association between open spaces and BMI *z*-score in a sample of 301 children (Crawford et al. 2010), another study found an inverse association between park density and attained BMI at 18 years of age in a sample of 3,173 children (Wolch et al. 2011). In another study of 3,831 children 3–16 years of age, Bell et al. (2008) found that higher greenness was significantly associated with lower BMI *z*-scores at time 2, and was also associated with lower odds of children increasing their BMI *z*-scores over 2 years, but found no cross-sectional or longitudinal association between residential density and BMI *z*-score. In a sample of 485 children from Kiel, Germany, Gose et al. (2013) found walkability, street type, socioeconomic status of the district, and perceived frequency of passing trucks/buses were associated with BMI standard deviation score over 4 years, but only neighborhood socioeconomic status had an effect on change in BMI standard deviation score.

There are a variety of potential explanations for our findings. In most cases, characteristics consistent with more walkable built environments were associated with lower BMI and some evidence of lower BMI change over time; this suggests that youth may be walking (or engaging in physical activity such as utilitarian physical activity) in built environments that promote energy expenditure. Supporting this, a recent review found that recreational open space has been associated with increased youth physical activity (Ding et al. 2011). Most studies included in this review found that increased land use mix was associated with higher youth physical activity—suggesting that people may walk more when there is a greater variety of land use or services available near one’s home (Ding et al. 2011).

To put the BMI *z*-score differences found in the current study in context, children living closest (quartile 1) to the nearest recreational open space had a lower cross-sectional BMI *z*-score (β = –0.06; 95% CI: –0.08, –0.03) compared with children living farthest away (quartile 4, reference), and a difference of 0.06 units in BMI *z*-score would translate to about a 0.3-kg or 0.6-lb difference in weight compared with an 11-year-old male (our population’s average age) with height, weight, and BMI at the 50th percentile for age and sex (Children’s Hospital of Philadelphia Research Institute 2013). Children with the least residential density (quartile 1) had a higher BMI *z*-score (β = 0.11; 95% CI: 0.08, 0.14) compared with children with the most residential density (quartile 4, reference): A difference in BMI *z*-score of 0.11 units is approximately 0.5-kg or 1-lb difference in weight compared with an average 11-year-old male (Children’s Hospital of Philadelphia Research Institute 2013). At the individual level, these differences might appear to be minimal; however, at the population level these differences might be more meaningful (Rose 1992).

The results did not change across models when specifying neighborhood median income in different ways. Although our findings sometimes changed direction from the unadjusted model to the fully adjusted model, this was due to adjustment for neighborhood median household income (which is strongly related to BMI and related to the walkable built environment exposures). However, adjusting for median household income is important to have an accurate understanding of the results. In sensitivity analyses, the findings by and large were maintained when stratified by quartiles of neighborhood median household income. However, in the results stratified by neighborhood median household income, significant cross-sectional associations seem to be isolated primarily in children in the sample from lower-income neighborhoods (mean Q1 = $48,435, mean Q2 = $72,626). Some other previous research has found that associations between built environment characteristics and childhood overweight/obesity existed predominantly in children in lower-income neighborhoods (Oreskovic et al. 2009a), perhaps suggesting that children in lower-income neighborhoods are more susceptible to the effects of their built environments. It is unclear why the direction of association changed in the unadjusted to the adjusted models including neighborhood median income; but the sample of children come from relatively high-income neighborhoods, and this might have influenced the findings.

This study has several limitations. First, GIS data quality is a concern in all GIS-based research; for example, sidewalk attributes may be less accurate in suburban environments. Second, our study design and conclusions may have been influenced by the neighborhood definition, and we assumed both that youth use their built environments (e.g., recreational open spaces) and spend sufficient time in residential neighborhoods for their effects to be meaningful (Kwan 2012a, 2012b). Indeed, the distance to open recreational space metric does not necessarily mean that the distance was walkable; persons near parks could drive to them (because we used the 15-km cutoff, many of these open spaces may be driven to). Furthermore, given the structure of the data, we were unable to account for other locations (e.g., schools) that might be salient to children—which could influence child physical activity/BMI. If children’s physical activity takes place mostly at school, or if they mostly access the “playing fields” from their schools, then the distance to the nearest open space from home (for example) will have less causal effect on their physical activity/BMI. Self-selection into neighborhoods (i.e., residential selection bias) could be a limitation, but we accounted for several demographic characteristics potentially associated with selection into neighborhoods; previous research suggests that this bias would attenuate associations (Boone-Heinonen et al. 2010). Because we used only the current address for each child, we were not able to incorporate residential trajectories into the analyses, which could result in exposure misclassification if the youth moved. We recognize that built environments can change, but they are posited to do so slowly (Duncan et al. 2011). Residual confounding is also possible (e.g., we could not account for household income because this information was not available in the electronic health records). All patients in the study had health insurance; our findings might not be generalizable to uninsured populations.

## Conclusions

Built environment characteristics that may increase walkability generally were associated with lower BMI *z*-scores in a large sample of children and adolescents from an electronic health record database, especially perhaps among children in lower-income neighborhoods. Thus, this study, using large electronic health record data, suggests that neighborhood physical features can affect BMI *z*-scores. Our findings suggest that modifying existing built environments to make them more walkable may reduce childhood obesity. Public policies at local and national levels likely should promote walkable built environments.

## Supplemental Material

(212 KB) PDF
