# Supplementary material for: Characteristics of Walkable Built Environments and BMI z-Scores in Children: Evidence from a Large Electronic Health Record Database
Source: Environ Health Perspect. 2014 Sep 23;122(12):1359–65. doi: 10.1289/ehp.1307704 (PMC4256697; doi:10.1289/ehp.1307704)
Supplement: (212 KB) PDF [file ehp.1307704.s001.508.pdf]

Environmental Health Perspectives doi:10.1289/ehp.1307704

Supplemental Material

Characteristics of Walkable Built Environments and BMI *z*-Scores in Children: Evidence from a Large Electronic Health Record Database

Dustin T. Duncan, Mona Sharifi, Steven J. Melly, Richard Marshall, Thomas D. Sequist, Sheryl L. Rifas-Shiman, and Elsie M. Taveras

Table S1. Associations of neighborhood walkability in quartiles with most recent, cross-sectional BMI *z*-score stratified by quartile of neighborhood household median income [β (95% CI)], N=49,770.

**Exposures**	**Q1 (12,429 – 63,229)**	**Q2 (63,291 – 81,378)**	**Q3 (81,481 – 108,646)**	**Q4 (108,750 – 218,667)**
Nearest recreational open space (km)				
Q1	-0.12 (-0.18,-0.05)*	-0.06 (-0.11, 0.00)	-0.04 (-0.09, 0.01)	-0.03 (-0.07, 0.02)
Q2	-0.08 (-0.15,-0.02)*	-0.04 (-0.09, 0.02)	-0.08 (-0.13,-0.03)*	-0.04 (-0.09, 0.01)
Q3	-0.04 (-0.11, 0.02)	-0.03 (-0.08, 0.03)	0.02 (-0.03, 0.07)	0.00 (-0.05, 0.04)
Q4	0.0 (ref)	0.0 (ref)	0.0 (ref)	0.0 (ref)
Trend *p*-value	<0.0001	0.04	0.01	0.10
Count of recreational open space				
Q1	0.12 (0.04, 0.20)*	0.17 (0.12, 0.23)*	0.11 (0.05, 0.16)*	0.06 (-0.01, 0.12)
Q2	0.11 (0.05, 0.18)*	0.13 (0.08, 0.19)*	0.21 (0.15, 0.27)*	0.08 (0.01, 0.14)*
Q3	0.05 (0.01, 0.09)*	0.17 (0.12, 0.22)*	0.06 (0.00, 0.11)	0.04 (-0.02, 0.11)
Q4	0.0 (ref)	0.0 (ref)	0.0 (ref)	0.0 (ref)
Trend *p*-value	<0.0001	<0.0001	<0.0001	0.12
Residential density				
Q1	0.12 (-0.02, 0.27)	0.10 (0.02, 0.18)*	0.17 (0.10, 0.23)*	0.11 (-0.01, 0.24)
Q2	0.12 (0.04, 0.20)*	0.12 (0.07, 0.18)*	0.14 (0.08, 0.20)*	0.11 (-0.02, 0.23)
Q3	0.13 (0.08, 0.17)*	0.09 (0.04, 0.14)*	0.06 (0.00, 0.13)	0.10 (-0.03, 0.24)
Q4	0.0 (ref)	0.0 (ref)	0.0 (ref)	0.0 (ref)
Trend *p*-value	<0.0001	<0.0001	<0.0001	0.28
Traffic density				
Q1	0.01 (-0.08, 0.10)	0.02 (-0.03, 0.08)	0.04 (-0.01, 0.09)	0.03 (-0.02, 0.08)
Q2	0.07 (0.02, 0.12)*	0.10 (0.05, 0.15)*	-0.01 (-0.06, 0.04)	0.03 (-0.02, 0.09)
Q3	0.01 (-0.04, 0.05)	0.07 (0.02, 0.12)*	0.00 (-0.06, 0.05)	0.02 (-0.03, 0.08)
Q4	0.0 (ref)	0.0 (ref)	0.0 (ref)	0.0 (ref)
Trend *p*-value	0.06	0.08	0.09	0.22
Average speed limit				
Q1	0.07 (0.01, 0.14)*	0.06 (0.00, 0.12)	0.00 (-0.05, 0.05)	0.02 (-0.03, 0.06)
Q2	-0.01 (-0.06, 0.04)	0.02 (-0.03, 0.07)	-0.02 (-0.07, 0.03)	0.01 (-0.04, 0.06)
Q3	0.01 (-0.04, 0.06)	0.01 (-0.04, 0.07)	-0.01 (-0.07, 0.04)	0.02 (-0.03, 0.07)
Q4	0.0 (ref)	0.0 (ref)	0.0 (ref)	0.0 (ref)
Trend *p*-value	0.13	0.04	0.88	0.49

Exposures	Q1 (12,429 – 63,229)	Q2 (63,291 – 81,378)	Q3 (81,481 – 108,646)	Q4 (108,750 – 218,667)
Sidewalk completeness				
Q1	0.07 (-0.04, 0.17)	0.05 (-0.01, 0.11)	0.05 (0.00, 0.10)	0.00 (-0.07, 0.07)
Q2	0.07 (0.01, 0.13)*	0.08 (0.02, 0.13)*	0.14 (0.09, 0.19)*	0.04 (-0.03, 0.11)
Q3	0.05 (0.01, 0.09)*	0.06 (0.01, 0.12)*	0.03 (-0.02, 0.09)	0.00 (-0.08, 0.08)
Q4	0.0 (ref)	0.0 (ref)	0.0 (ref)	0.0 (ref)
Trend *p*-value	0.01	0.06	0.01	0.72
Intersection density				
Q1	0.11 (-0.02, 0.23)	0.05 (-0.02, 0.11)	0.08 (0.02, 0.13)*	0.08 (0.00, 0.16)
Q2	0.10 (0.03, 0.16)*	0.10 (0.05, 0.15)*	0.12 (0.06, 0.17)*	0.07 (-0.01, 0.15)
Q3	0.06 (0.01, 0.10)*	0.05 (0.00, 0.10)	-0.01 (-0.07, 0.04)	0.06 (-0.03, 0.14)
Q4	0.0 (ref)	0.0 (ref)	0.0 (ref)	0.0 (ref)
Trend *p*-value	0.001	0.01	<0.0001	0.07
Land use mix				
Q1	0.08 (-0.01, 0.16)	0.07 (0.02, 0.12)*	0.04 (-0.01, 0.09)	0.03 (-0.02, 0.08)
Q2	0.07 (0.01, 0.13)*	0.06 (0.01, 0.12)*	0.03 (-0.02, 0.08)	0.02 (-0.05, 0.08)
Q3	0.04 (0.00, 0.09)	0.09 (0.04, 0.14)*	0.04 (-0.02, 0.10)	0.02 (-0.05, 0.09)
Q4	0.0 (ref)	0.0 (ref)	0.0 (ref)	0.0 (ref)
Trend *p*-value	0.01	0.02	0.16	0.32

Notes: For analyses, we categorized each walkable built environment variable into quartiles, so each built environment variable is divided into four equal groups. For example, those in quartile 1 of the nearest recreational open space have closer open spaces compared to quartile 4. Models adjusted for age, gender, race/ethnicity (with a missing category), and neighborhood median household income (continuous). Significant findings are noted by an *.

Table S2. Associations of neighborhood walkability in quartiles with change in BMI *z*-score stratified by quartile of neighborhood household median income [β (95% CI)], N=46,813.

Exposures	Q1 (12,429 – 63,229)	Q2 (63,291 – 81,378)	Q3 (81,481 – 108,646)	Q4 (108,750 – 218,667)
Nearest recreational open space (km)				
Q1	-0.04 (-0.08, 0.01)	-0.03 (-0.07, 0.00)	-0.03 (-0.07, 0.00)	0.03 (0.00, 0.06)
Q2	-0.01 (-0.05, 0.03)	-0.01 (-0.05, 0.02)	-0.03 (-0.06, 0.00)	0.03 (0.00, 0.06)
Q3	0.01 (-0.04, 0.05)	0.00 (-0.04, 0.03)	0.00 (-0.04, 0.03)	0.02 (-0.01, 0.05)
Q4	0.0 (ref)	0.0 (ref)	0.0 (ref)	0.0 (ref)
Trend *p*-value	0.03	0.06	0.02	0.03
Count of recreational open space				
Q1	0.06 (0.00, 0.11)	0.02 (-0.02, 0.06)	0.07 (0.03, 0.11)*	0.00 (-0.04, 0.05)
Q2	0.02 (-0.02, 0.06)	0.01 (-0.02, 0.05)	0.04 (0.00, 0.08)	0.03 (-0.02, 0.08)
Q3	0.02 (-0.01, 0.05)	0.02 (-0.02, 0.05)	0.04 (0.00, 0.08)	0.02 (-0.03, 0.06)
Q4	0.0 (ref)	0.0 (ref)	0.0 (ref)	0.0 (ref)
Trend *p*-value	0.03	0.41	0.0004	0.67
Residential density				
Q1	0.03 (-0.06, 0.13)	-0.01 (-0.07, 0.04)	0.08 (0.04, 0.13)*	0.08 (-0.01, 0.17)
Q2	0.02 (-0.03, 0.08)	0.02 (-0.02, 0.05)	0.07 (0.02, 0.11)*	0.06 (-0.03, 0.16)
Q3	0.03 (-0.01, 0.06)	-0.01 (-0.04, 0.03)	0.05 (0.01, 0.10)*	0.10 (0.00, 0.20)
Q4	0.0 (ref)	0.0 (ref)	0.0 (ref)	0.0 (ref)
Trend *p*-value	0.15	0.64	0.0003	0.47
Traffic density				
Q1	-0.01 (-0.08, 0.05)	0.04 (0.00, 0.07)	0.05 (0.01, 0.08)*	0.01 (-0.03, 0.04)
Q2	0.02 (-0.02, 0.05)	0.04 (0.00, 0.07)	0.01 (-0.03, 0.05)	0.02 (-0.02, 0.05)
Q3	0.00 (-0.03, 0.03)	0.02 (-0.01, 0.06)	0.02 (-0.02, 0.06)	0.05 (0.00, 0.09)
Q4	0.0 (ref)	0.0 (ref)	0.0 (ref)	0.0 (ref)
Trend *p*-value	0.74	0.04	0.01	0.66
Average speed limit				
Q1	0.01 (-0.03, 0.06)	0.02 (-0.02, 0.06)	0.01 (-0.02, 0.05)	0.03 (0.00, 0.06)
Q2	0.00 (-0.03, 0.04)	-0.01 (-0.05, 0.03)	-0.02 (-0.06, 0.01)	0.01 (-0.03, 0.05)
Q3	0.00 (-0.04, 0.03)	0.00 (-0.04, 0.03)	0.00 (-0.04, 0.03)	0.01 (-0.03, 0.04)
Q4	0.0 (ref)	0.0 (ref)	0.0 (ref)	0.0 (ref)
Trend *p*-value	0.49	0.43	0.61	0.09

Exposures	Q1 (12,429 – 63,229)	Q2 (63,291 – 81,378)	Q3 (81,481 – 108,646)	Q4 (108,750 – 218,667)
Sidewalk completeness				
Q1	0.04 (-0.03, 0.11)	0.06 (0.02, 0.10)*	0.06 (0.02, 0.09)*	0.01 (-0.04, 0.06)
Q2	0.02 (-0.02, 0.06)	0.03 (0.00, 0.07)	0.06 (0.02, 0.10)*	0.01 (-0.04, 0.06)
Q3	0.02 (-0.01, 0.05)	0.05 (0.01, 0.08)*	0.02 (-0.02, 0.06)	0.03 (-0.03, 0.08)
Q4	0.0 (ref)	0.0 (ref)	0.0 (ref)	0.0 (ref)
Trend *p*-value	0.13	0.01	0.0004	0.94
Intersection density				
Q1	-0.05 (-0.13, 0.04)	0.02 (-0.02, 0.06)	0.06 (0.02, 0.10)*	0.06 (0.00, 0.12)
Q2	0.02 (-0.03, 0.06)	0.00 (-0.03, 0.04)	0.04 (0.00, 0.08)	0.07 (0.01, 0.13)*
Q3	0.01 (-0.03, 0.04)	0.02 (-0.02, 0.05)	0.01 (-0.03, 0.05)	0.07 (0.01, 0.13)*
Q4	0.0 (ref)	0.0 (ref)	0.0 (ref)	0.0 (ref)
Trend *p*-value	0.92	0.73	0.0001	0.48
Land use mix				
Q1	-0.01 (-0.07, 0.05)	0.02 (-0.01, 0.06)	0.04 (0.00, 0.07)	-0.02 (-0.05, 0.02)
Q2	0.01 (-0.03, 0.05)	-0.02 (-0.06, 0.01)	0.05 (0.01, 0.09)*	-0.02 (-0.06, 0.03)
Q3	0.00 (-0.03, 0.02)	0.01 (-0.02, 0.05)	-0.01 (-0.05, 0.03)	-0.02 (-0.07, 0.04)
Q4	0.0 (ref)	0.0 (ref)	0.0 (ref)	0.0 (ref)
Trend *p*-value	0.95	0.52	0.01	0.49

Notes: For analyses, we categorized each walkable built environment variable into quartiles, so each built environment variable is divided into four equal groups. For example, those in quartile 1 of the nearest recreational open space have closer open spaces compared to quartile 4. Models adjusted for age, change in age baseline-follow-up, gender, race/ethnicity (with a missing category), and neighborhood median household income (continuous). Significant findings are noted by an *.
